# Attitudes of registered nurses about the end – of – life care in multi-profile hospitals: a cross sectional survey

**DOI:** 10.1186/s12904-020-00637-7

**Published:** 2020-08-19

**Authors:** Aurelija Blaževičienė, Lina Laurs, Jamesetta A. Newland

**Affiliations:** 1 Department of Nursing and Care, Lithuanian University of Health Sciences, Eiveniu 4, 44307 Kaunas, LT Lithuania; 2 New York University Rory Meyers College of Nursing, New York, NY USA

**Keywords:** Registered nurses, End-of-life care, Barriers to care, Facilitating behaviors

## Abstract

**Background:**

End-of-life care is provided in a variety of healthcare settings, not just palliative care hospitals. This is one reason why it is very important to assess all barriers to end-of-life care and to provide safe and quality services to patients. This study was aimed at describing nurses’ attitudes in providing end-of-life care and exploring barriers and facilitating behaviors of nurses in multi-profile hospitals in Eastern Europe.

**Methods:**

A descriptive, correlational design was applied in this study, using a cross-sectional survey of 1320 registered nurses within 7 hospitals in Lithuania.

**Results:**

Registered nurses working in the three different profiles emphasized safe and effective care and the importance of meeting the patient’s spiritual needs at the end of life. The main barriers assigned by nurses caring for patients at the end of life were angry family members, inadequate understanding of nursing care by the patient’s relatives; lack of time to talk to patients, lack of nursing knowledge to deal with the bereaved patient’s family, lack of evaluation of nurses’ opinions, and the evasion by physicians to talk about the diagnosis and their over-optimistic view of the situation. The main facilitating behaviors to improve nursing care were end-of-life training, volunteering, and family involvement.

**Conclusions:**

Spiritual needs were identified by nurses as the primary needs of patients at the end of life. Family-related barriers remain one of the main barriers to end-of-life care. Also, the behavior of physicians and their relationship with nurses remains one of the most sensitive issues in end-of-life care.

## Background

The National Cancer Institute describes end-of-life care as “care given to people who are near the end of life and have stopped treatment to cure or control their disease. End-of-life (EOL) care includes physical, emotional, social, and spiritual support for patients and their families.” End-of-life care is a portion of palliative care that is directed toward the care of persons who are nearing end of life [1]. Palliative care is fundamental to health and human dignity and is a basic human right. Palliative care staff have specialist expertise in symptom management; and emotional, spiritual, practical, and cultural care. They might be involved in managing more complex care problems [2].

Nurses, physicians, and allied health professionals agree that EOL care should be provided to patients in palliative care units or hospitals where staff has sufficient knowledge of EOL care [2]. However, EOL care is provided in a variety of healthcare settings, not just palliative care hospitals, which makes it very important to assess all the barriers to EOL care and to provide safe and quality services to patients. Therefore, providing EOL care in any setting can be challenging [3]. Nurses play a key role in EOL care, and their approach to patient EOL care and preparedness is an important factor in ensuring quality patient and family-centered care [4, 5]. End-of-life and palliative care provide practical help with daily tasks as well. The goal is to improve quality of life for patients, family, friends, and caregivers. End-of-life and palliative care are based on what the patient needs [6].

The attitudes of nurses towards death and nurses’ readiness to provide EOL care might influence the care they provide to terminal or dying patients [7]. Factors that determine attitudes towards death and dying depend not only on culture, society, values orientation, and religion but also on an individual’s perception and personal attitudes of death and dying [8]. Patients’ deaths often lead to anxiety and undesirable attitudes among nurses, which can influence the quality of patient care. Communication with palliative and terminally ill patients might be reflected by a nurse’s attitude. Therefore, the quality of care is highly dependent on the professional readiness of both nurses and physicians to provide EOL care. Many nurses will not have experiences of meeting or caring for someone who is dying. As death can occur in any setting at any time, it is vital that all registered nurses regardless of the setting in which they work, have EOL care training [9].

Sasahara et al. revealed that 92% of the nurses expressed concerns about providing EOL care, and it was particularly difficult for them to help patients express their anger and concern regarding death. And 91% of the nurses did not know how to react when a patient start talking about death and dying [10]. In general, nurses felt discomfort when talking about EOL issues with patients and their loved ones. Based on the scientific literature, this trend is similar in many cultures [8, 11, 12]. Researchers revealed that nurses did not feel ready to discuss EOL issues with patients because EOL care was emotionally distressing and required a lot of specific knowledge [8]. Therefore, communication with patients was hard work, and nurses expressed a desire to do something else instead [13].

Researchers emphasized that positive nurses’ attitudes in caring for dying patients could be influenced by nurses’ demographic characteristics, experience, and previous education. Nurses with greater experience in dealing with dying patients felt more confident and had a more positive attitude in providing EOL care [14, 15]. In addition, nurses’ clinical experience and time spent with dying patients increased positive attitudes toward EOL care [16, 17]. It is important to explore nurses’ attitudes toward caring for dying patients and to develop strategies to alleviate these communication difficulties between nurses and patients to improve care in the terminal phase. Therefore, the importance of EOL nursing care underscores the necessity to investigate nurses’ attitudes and their readiness to provide EOL care in multi-profile hospitals.

### Research context

In Lithuania, palliative care is provided in an institution, a day centre, or at the patient’s home. Palliative care is the comprehensive care of patients with incurable, progressive diseases. Depending on the needs of the patient and his or her family members, necessary assistance is provided to the patient by a doctor, nurse, social worker, psychologist, and other staff. Palliative care was introduced as a concept in 2006 under the National Cancer Control and Prevention Programme. Legal regulation of EOL issues in Lithuania started in 2007; the procedure for providing palliative care was approved. Regulatory arrangements for palliative care provision under contracts with the National Health Insurance Fund (NHIF) were introduced in 2007. The contract terms included a description of indications for referral, relevant procedures and provision standards (e.g., a team of at least three professionals, including a physician, nurse, and social worker; a list of equipment for health-care facilities; minimum duration of consultations at a patient’s home) [18].

Subsequently, in 2012 a description of the requirements for the provision of supportive treatment and nursing services was approved. Following these descriptions, EOL care was provided. However, writing a last will and testament of the future is still not standard practice, and discussion with the patient and his or her relatives about EOL care priorities remains a forbidden topic. In most cases, health care professionals must take moral responsibility for decisions related to EOL care. Also, the reality is that intersectoral collaboration for health still remains a delicate issue in Lithuania [19, 20]. Regardless of the general acceptance that other sectors of society are important for the health of the population, no effective mechanisms to implement this intersectoral collaboration have been in place until recently [19–21]. And cross-sectoral and multidisciplinary collaboration in the provision of not only life care services but also all other health care services is essential to ensure quality and safe services for the patient. The aim of this study was to describe nurses’ attitudes in providing EOL care and exploring barriers and facilitating behaviors of nurses in multi-profile hospitals in Eastern Europe.

## Methods

### Research design

A descriptive, cross-sectional, correlational design was applied in this study.

### Sample

Registered nurses (RNs) were recruited from all unit types and specialities (surgical, therapeutic, and intensive care), working in the seven large municipal multi-profile hospitals representing Lithuania. In Lithuania, there are approximately 22,500 RNs, and 2560 work in these hospitals in all unit types. According to a sample calculation formula, 378 nurses (22,500 nurses in Lithuania, 95% probability, and 0.05 error percentage) were needed for the study [22]. To account for not all voluntarily consenting to participate, a higher sample size was targeted to be adequately powered. For this study, 1310 RNs were selected to participate. This population of RNs served as the same pool from which a different sample was drawn for another study [23]. Nurses who worked day, night, or mixed shifts during the study were invited to participate in the study. The study did not include nurses who were on maternity or annual leave and had a certificate of incapacity for work.

### Instruments

Nurses’ readiness to care for patients at the EOL and attitudes toward their care were assessed using the Questionnaire of Helps and Obstacles in Providing End-of-Life Care to Dying Patients and Their Families [24]. The questionnaire was validated and verified in a previous study conducted by the authors [25]. Respondents were able to choose an option according to a Likert scale with 1 = no help/not an obstacle to 5 = extremely intense help/extremely large obstacle. Socio-demographic characteristics, such as age, gender, employment, current workplace, and length of current employment were also collected.

Cronbach’s alpha for the questionnaire was established at 0.86, meeting the requirement for acceptance. Similar questionnaires have been used in studies with oncology departments in Lithuania and intensive care departments in Spain and the United States [25–27].

### Data collection

Questionnaires were distributed to nurses (face-to-face) by one of the authors at the hospitals during the months of September to November 2017. During the study, 1320 questionnaires were distributed; 1180 questionnaires were returned, of which 1055 were satisfactorily completed (response rate 79.9%).

### Data analysis

Survey data were analyzed using SPSS for Windows 19.0 (SPSS Statistics for Windows) [28]. Only fully completed questionnaires were used for analysis. The level of significance selected for testing data points was established at *p* ≤ 0.05. Descriptive statistics were used to calculate the average values of the variables within a 95% confidence interval; standard deviation of the scores were also calculated.

### Ethical considerations

The study was approved by the Bioethics Committee at the Lithuanian University of Health Sciences (No. BE- 2-27). Hospital administrations were informed of the research goals. Verbal informed consent was obtained from each participant following an explanation of the research study goals during the face-to-face recruitment process. This process was approved by the ethics committee. Nurses had the right to refuse participating in the study or withdraw at any time without penalty. The confidentiality of participants was assured, and anonymity was maintained. All data were summarized and reported only in the aggregate.

## Results

### Sample characteristics

Sociodemographic characteristics of 1055 RNs are described in Table 1. The average age of RNs participating in the study was 45.8 ± 9.9 years, and the average length of service was 23.4 ± 11.1 years. The majority of respondents were married (*n* = 668, 63.3%) and worked part-time (*n* = 786, 74.5%) in mixed shifts (*n* = 716, 67.9%). Almost half had completed medical college (*n* = 495, 46.9%). Of all RNs, 49% (*n* = 516) worked in the internal medicine department, with 32.6% (*n* = 344) in the surgery department, and 18.4% (*n* = 195) in the intensive care department.

**Table 1:** Sample Characteristics

Characteristic	***N***	%	Hospitals by Region ***n*** (%)						
			Kaunas	Klaipeda	Panevėžys	Alytus	Marijampolė	Vilnius	Šiauliai
**Age, years**									
to 44 years	349	33.1	30 (29.4)	26 (26.8)	85 (30.6)	11 (12.8)	15 (22.4)	79 (51.0)	103 (38.1)
45 to 50 years	374	35.5	38 (37.3)	30 (30.9)	100 (36.0)	32 (37.2)	32 (47.8)	49 (31.6)	93 (34.4)
51 years and older	332	31.5	34 (33.3)	41 (42.3)	93 (33.5)	43 (50.0)	20 (29.9)	27 (17.4)	74 (27.4)
**Educational preparation**									
Higher University Education	115	10.9	15 (14.7)	21 (21.6)	24 (8.6)	3 (3.5)	6 (9.0)	27 (17.4)	19 (7.0)
College	940	89.1	87 (85.3)	76 (78.4)	254 (91.4)	83 (96.5)	61 (91.0)	128 (82.6)	251 (93.0)
**Department/ Unit**									
Surgery department	344	32.6	27 (26.5)	31 (32.0)	79 (28.4)	17 (19.8)	24 (35.8)	79 (51.0)	87 (32.2)
Intensive Care department	195	18.5	14 (13.7)	16 (16.5)	53 (19.1)	18 (20.9)	8 (11.9)	44 (28.4)	42 (15.6)
Internal Medicine	516	48.9	61 (59.8)	50 (51.5)	146 (52.5)	51 (59.3)	35 (52.2)	32 (20.6)	141 (52.2)
**Shift**									
Morning	209	19.8	21 (20.6)	13 (13.4)	61 (21.9)	10 (11.6)	9 (13.4)	32 (20.6)	63 (23.3)
Night/Afternoon shift	130	12.3	21 (20.6)	0 (0)	36 (12.9)	6 (7.0)	18 (26.9)	9 (5.8)	40 (14.8)
Mixed (morning, afternoon, and night shift)	716	67.9	60 (58.8)	84 (86.6)	181 (65.1)	70 (81.4)	40 (59.7)	114 (73.5)	167 (61.9)
**Years experience in nursing**									
0 to 5	114	10.8	16 (15.7)	14 (14.4)	25 (9)	0 (0)	2 (3.0)	20 (12.9)	37 (13.7)
6 to 15	143	13.6	12 (11.8)	7 (7.2)	33 (11.9)	3 (3.5)	4 (6.0)	38 (24.5)	46 (17.0)
16 to 25	273	25.9	25 (24.5)	15 (15.5)	75 (27.0)	18 (20.9)	19 (28.4)	50 (32.3)	71 (26.3)
26 to 31	272	25.8	22 (21.3)	24 (24.7)	76 (27.3)	31 (36.0)	26 (38.8)	33 (21.3)	60 (22.2)
> 31	253	24.0	27 (26.7)	37 (38.1)	69 (24.9)	34 (39.5)	16 (23.9)	14 (9.0)	56 (20.7)

### Registered nurses’ attitudes to EOL care

According to the study, RNs working in the three different profiles emphasized safe and effective care for patients at the EOL. RNs also emphasized the importance of meeting the patient’s spiritual needs in EOL care, i.e., the patient should have the right to a dignified and painless death. The survey revealed a statistically significant difference between RNs in the three departments in attitudes about working with seriously ill patients who frequently died. RNs in the surgical department more than those in the intensive care and internal medicine departments felt these nurses required the help of a psychologist (*M* = 4.20, *p* = .009). The RNs in the surgical departments also indicated stronger attitudes that family and relatives should not be limited in time and duration of the patient visit (*M* = 4.16, *p* < .001). Meanwhile, RNs working in the internal medicine departments were more likely to say that patients should not be permanently suppressed by sedation drugs (*M* = 3.69, *p* = < .001). And RNs working in intensive care departments felt most psychologically prepared to deal with the problems at the EOL (*M* = 3.67, *p* = .011) (Table 2).

**Table 2:** RN attitudes toward patient care at the end-of-life depending on the department

Row. No.	Statement	Surgical department	Intensive care department	Internal medicine department	*N* = 1055
		*M* (*SD*)	*M* (*SD*)	*M* (*SD*)	*p*
		*n* = 344	*n* = 195	*n* = 516	
1	The patient should continue to receive all interventions to prevent pressure sores	4.66 (0.59)	4.73 (0.53)	4.68 (0.51)	0.331^c^
2	The patient is entitled to a dignified and painless death	4.62(0.60)	4.69 (0.54)	4.67 (0.56)	0.392^c^
3	The patient should always be given the opportunity to receive last rituals that are appropriate to the religious and spiritual beliefs of the patient and their family	4.60 (0.58)	4.62 (0.62)	4.60 (0.58)	0.839^c^
4	The patient should be cared for in the privacy of a private room	4.55 (0.60)	4.56 (0.67)	4.54 (0.63)	0.659^c^
5	During EOL care, oro/endotracheal suction should be continued to maintain the airway of the patient	4.45 (0.60)	4.44 (0.67)	4.42 (0.60)	0.629^c^
6	Healthcare professionals working with patients with extremely serious conditions and frequent deaths, need psychological help	4.20 (0.79)	4.05 (0.83)	4.01 (0.89)	0.009^c^
7	The family and friends of the patient should be permitted to visit at any time, day or night	4.16 (1.04)	3.28 (1.25)	4.04 (1.09)	< 0.001^c^
8	It is advisable for a patient suffering from an incurable disease to be given the optimum amount of painkillers, despite the fact that this would accelerate his death	4.14 (0.78)	4.21 (0.76)	4.20 (0.78)	0,419^c^
9	Patients have the right to refuse treatment, even though this would result in their death	3.87 (0.93)	3.86 (0.97)	3.95 (0.91)	0.354^c^
10	Some patients may be excluded from their treatment and nursing decisions because of doubts about their ability to assess the situation	3.86 (0.78)	3.92 (0.80)	3.83 (0.83)	0.481^c^
11	Talking with doctors about solving end-of-life problems in a patient has a positive effect on nurses’ job satisfaction	3.76 (0.94)	3.91 (0.86)	3.73 (0.98)	0.128^c^
12	During EOL care, the patient should continue to receive fluids to maintain hydration	3.72 (1.04)	3.87 (1.04)	3.70 (1.03)	0.126^c^
13	Nurses have sufficient knowledge of their patients to make an informed decision about what they want	3.67 (0.97)	3.62 (0.97)	3.71 (1.01)	0.393^c^
14	Interviews with the patient’s family about solving the patient’s end-of-life problems have a positive influence on nurses’ job satisfaction	3.64 (1.00)	3.66 (0.95)	3.59 (1.01)	0,730^c^
15	Patient consciousness should not be permanently suppressed by sedation	3.52 (1.01)	3.27 (1.06)	3.69 (1.01)	< 0.001^c^
16	You feel psychologically prepared to deal with critical care issues	3.49 (1.01)	3.67 (1.05)	3.66 (0.97)	0.011^c^
17	Nurses must respect the patient’s wishes, even if they are contrary to their own beliefs	3.32 (1.01)	3.29 (1.09)	3.32 (1.06)	0.941^c^
18	The family and friends of the patient should be permitted to visit the patient at the bedside without a restriction on the number of family members and friends	2.92 (1.19)	2.63 (1.17)	2.91 (1.24)	0.006^c^

Used Kruskal Wallis Test

### Registered nurses’ attitudes to barriers and facilitating behaviors in providing EOL care

Analyzing the most common barriers to EOL care, the survey data indicated that for the first block of barriers, with a comparable average of more than 4 points, the respondents in all departments attributed dealing with angry family members as a barrier (*p* = .004) and inadequate understanding of the nursing care by the patient’s relatives (NS). RNs working in intensive care departments were more likely to identify the barrier of family and friends who regularly called the nurse to find out about the patient’s condition rather than listening to informed family members (*M* = 4.02, *p* = .034). Also, for intensive care RNs, family members disagreeing on the kind of care that was most adequate for the patient was perceived as a barrier (*M* = 3.90, *p* = .046).

For the second block of barriers, with a mean score of 4 to 3.5, RNs assigned family members’ inadequate knowledge about the situation and lack of time to talk to patients about their wishes for EOL problems as barriers. And in the third block of barriers, with scores of less than 3.5, RNs attributed the lack of knowledge to communicate with the bereaved patient’s family, the lack of evaluation of nurses’ opinions, and the evasion of physicains to talk about diagnosis and their over-optimistic view of the situation as barriers (Table 3). Group differences were not statistically significant.

**Table 3:** RN attitudes to potential barriers in ensuring patient care at end-of-life depending on the department

Row. No.	Statement	Surgical department *n* = 344	Intensive care department *n* = 195	Internal medicine department *n* = 516	*N* = 1055
		*M* (*SD*)	*M* (*SD*)	*M* (*SD*)	*p*
1	The patient’s relatives having inadequate understanding of the situation interfere with the nurses’ duties	4.16 (0.73)	4.09 (0.75)	4.18 (0.72)	0.319^c^
2	Nurses have to deal with angry patient’s family members	4.04 (0.86)	4.05 (0.87)	4.18 (0.87)	0.004^c^
3	Family has no access to psychological help after being informed about the patient’s diagnosis	3.97 (0.91)	4.04 (0.89)	3.86 (1.04)	0.217^c^
4	Usually there is no time for conversations with patients about their wishes concerning the end of life decisions	3.95 (0.85)	3.89 (0.87)	4.00 (0.87)	0.150^c^
5	Family members or friends regularly call for a nurse in order to find out about the patient’s condition instead of addressing an informed family member	3.83 (0.87)	4.02 (0.85)	3.83 (0.92)	0.034^c^
6	Very often, the patient’s family members disagree on which treatment is most appropriate.	3.80 (0.81)	3.75 (0.89)	3.83 (0.85)	0.449^c^
7	The patient’s family members disagree on what kind of care is the most adequate	3.72 (0.87)	3.90 (0.78)	3.85 (0.87)	0.046^c^
8	The lack of nursing knowledge on how to treat the patient’s grieving family	3.42 (0.99)	3.33 (0.99)	3.26 (1.06)	0.125^c^
9	The nurse’s opinion on immediate patient care is not welcome, valued or discussed	3.40 (1.09)	3.46 (1.08)	3.39 (1.11)	0.770^c^
10	Physicians are too optimistic about the patient’s survival prospects during conversations with the patient’s family members	3.27 (0.99)	3.19 (0.97)	3.25 (1.03)	0.627^c^
11	Physicians are evasive and avoid conversation with the patient and/or family members	3.09 (1.10)	2.91 (1.07)	3.08 (1.14)	0.251^c^

Used Kruskal Wallis Test

Data for analyzing factors that would facilitate EOL care are presented in Table 4. RNs across all departments indicated that patient family education on how to deal with the seriously ill would facilitate the work of nurses. Similarly, nurses working in all three departments said that EOL training, volunteering, and family involvement would facilitate EOL care. Group differences were not statistically significant.

**Table 4:** Factors facilitating end-of-life care for patients depending on the department

Row. No.	Statement	Surgical department *n* = 344	Intensive care department *n* = 195	Internal medicine department *n* = 516	*N* = 1055
		*M* (*SD*)	*M* (*SD*)	*M* (*SD*)	*p*
1	Teaching families how to act with a dying patient	4.08 (0.79)	4.15 (0.65)	4.18 (0.67)	0.343^c^
2	End of life patient care training	3.97 (0.80)	3.97 (0.67)	4.06 (0.75)	0.066^c^
3	Auxiliary personnel helping the nurse with the patient’s care	3.74 (0.86)	3.66 (0.90)	3.80 (0.93)	0.130^c^
4	Having one family member be the designated contact person for all other family members regarding information about the patient.	3.66 (1.05)	3.87 (0.92)	3.75 (1.03)	0.131^c^
5	The family of the patient who appreciates your work in caring for a patient with a serious condition	3.61 (0.91)	3.58 (0.86)	3.55 (0.99)	0.736^c^
6	Nurse talking with patient about their feelings and thoughts about death	3.47 (0.93)	3.60 (0.88)	3.49 (0.97)	0.263^c^

Used Kruskal Wallis Test

## Discussion

The quality of care for dying patients is determined by the nurses’ attitude towards the end of life. The RNs who participated in this study stated that it was very important to meet the patient’s spiritual needs. This is also highlighted by research data from other researchers [29, 30]. Researchers found that patients in the terminal stages faced not only physical but also spiritual difficulties; they wanted to deal with their spiritual concerns with nurses or other health care staff [31, 32]. A holistic approach to terminal patient care is essential for EOL care, and spirituality in nursing is an important element of holistic care. Most EOL interventions focus predominantly on symptom control, rather than holistic care [31]. Data presented from this study revealed that nurses had a holistic approach to EOL patient care. They emphasized the importance not only of safe and effective care but also that the patient should have the right to a dignified and painless death and the last religious ritual should be provided.

Exploring facilitating behaviors towards EOL care from the perspective of nurses may lead to better understanding barriers to EOL care. Several studies have revealed that the main barriers to EOL care were patients’ relatives, who were inadequately judgmental or angry, and physician behavior [26, 33, 34]. In this study, RNs in all wards also identified patients’ relatives, communication with relatives, and relatives’ reluctance to accept poor prognosis as major barriers to care. A second set of factors attributed by the nurses in this study that made EOL care difficult was lack of time to talk with patients about their preferences for EOL care. Caring for seriously ill patients requires the nurse to spend more time addressing the patient’s physical needs, leaving less time to attend to the patient’s and family’s spiritual and psychological needs. The family’s expectations of improvement were also not realistic for reasons noted.

Clinical factors, taking into account the patient’s values, should be considered when continuing aggressive care, continuing therapy, or discontinuing life supportive measures. These solutions are complex and differ widely across cultures [11, 12]. Doctors play a key role in decisions to start, continue, or stop care. And one of the important barriers for nurses was that their views on direct patient care were unwanted, undervalued, or irrelevant. One older research study suggested that physicians in Northern and Central Europe were more likely to discuss EOL care with intensive care unit nurses than physicians in the rest of Europe, North America, Japan, or Brazil [35]. Diverging attitudes of nurses and physicians towards EOL care could also be a serious barrier to providing quality care [24]. But have attitudes really changed? Change was confirmed by the data from this study. Nurses identified one of the main barriers to EOL care was that nurses’ opinions on immediate patient care was not welcome, valued or discussed, and as a result both patient dissatisfaction with services and nurses’ dissatisfaction with work were prominent.

Analyzing what behaviors would support and improve EOL care, RNs in this study identified that patient family education on how to treat a seriously ill patient, as well as volunteers to help nurses and evaluate the work of the nurse, would greatly facilitate and improve care. End-of-life care would be facilitated by sufficient time for family members to say goodbye to the deceased, assistance from social workers or volunteers in providing care, having family members accept that patients are dying, and time to spend on emotions [36, 37].

## Conclusion

Nurses’ attitudes towards dying EOL patient care might depend on the departments where they work in clinical practice. Addressing spiritual needs was identified by nurses as the primary need of patients at the end of life. Barriers to EOL care, as percived by RNs, still exist. Family-related barriers remained one of the main barriers to EOL care. Also, the behavior of physicians and their relationships with nurses remained one of the most sensitive issues in EOL care. Based on the current identified barriers, recommendations for possible areas of focus might include: 1) family education and inclusion in EOL care; 2) collaboration between physicians and nurses in EOL decisions for patients; and 3) creating an appropriate work environment that relieves the psychological burden for both family members and care givers.

### Relevance to clinical practice

The results of this study draw attention to the need for family education and inclusion in the patient’s EOL care. Collaboration between physicians and nurses related to EOL decisions for patients is important. And creating an appropriate work environment that relieves the psychological burden of both relatives and care givers should be implemented in clinical practice; this change might ensure improved quality and safety of care for patients.

### Research strength and limitation

A number of studies have been conducted that analyzed the attitudes of nurses working in oncology or palliative care departments towards death and their readiness to provide care to patients at the end of life. However, very few studies have evaluated the attitudes of RNs working in various clinical units towards death and readiness to provide EOL care. The current study allows nurse researchers to anticipate the prevailing trend in multi-profile hospitals in Eastern Europe and to form further, in-depth research in this area. There were limitations of the study. Prior training on EOL issues of the RNs who participated in the study was not evaluated. It is not known whether as part of their undergraduate or postgraduate courses, any or all healthcare professionals receive training on EOL care and related factors in order to provide patients with quality services not only in oncology or palliative care units but also in the multi-profile hospitals. Only RNs working in the seven hospitals were invited to participate in this study. The attitudes and experiences of nurses working in community or other health care settings might be different from those of nurses in the hospital. An expanded study that includes physicians and other staff as participants would provide a deeper insight into how health care professionals work together to provide quality safe EOL care.

## Data Availability

The datasets used and/or analyzed during the current study are available from the corresponding author upon reasonable request.

## Abbreviations

EOL: End of life
RN: Registered nurse
